# The zebrafish lens proteome during development and aging

**Published:** 2009-11-13

**Authors:** Teri M.S. Greiling, Scott A. Houck, John I. Clark

**Affiliations:** 1 Department of Biological Structure, University of Washington, Seattle WA; 2 Department of Ophthalmology, University of Washington, Seattle WA

## Abstract

**Purpose:**

Changes in lens protein expression during zebrafish development results in a smooth gradient of refractive index necessary for excellent optical function. Age-related changes in crystallin expression have been well documented in mammals but are poorly understood in the zebrafish.

**Methods:**

In the zebrafish lens, a systematic analysis of protein content with age was performed using size exclusion chromatography (SEC) combined with linear trap quadrupole Fourier transform tandem mass spectrometry (LTQ-FT LC-MS/MS; rank-order shotgun) proteomics in lenses of larval, juvenile, and adult zebrafish.

**Results:**

α-Crystallins, previously shown to have low abundance in the zebrafish lens, were found to increase dramatically with maturation and aging. SEC determined that β-crystallin was predominant at 4.5 days. With age, the α- and γ-crystallins increased, and a high molecular weight fraction appeared between six weeks and six months to become the dominant component by 2.5 years. Similarly, shotgun proteomics determined that β-crystallins were the predominant proteins in the young lens. With age, the proportion of α- and γ-crystallins increased dramatically. After crystallins, calpain 3, membrane, and cytoskeletal proteins were most abundant. Five new β-crystallins and 13 new γ-crystallins were identified.

**Conclusions:**

As expected, SEC and proteomics demonstrated changing levels of protein expression with age, especially among the crystallins. The results also confirmed the existence of novel crystallins in the zebrafish genome.

## Introduction

Lens crystallins are proteins expressed at high concentrations in lens cells to achieve the high index of refraction required for normal optical function. Crystallin proteins are organized in short-range, glass-like order in the cytoplasm and are vital for the development and maintenance of lens transparency [1-3]. α-Crystallins, members of the small-heat shock protein family, protect against lens opacity by preventing the aggregation of unfolding proteins and maintaining cytoskeletal organization [4-8]. Similarly, mutations in α-, β-, or γ-crystallins have been linked to loss of transparency and human congenital cataract formation [9,10].

The expression levels of different crystallins vary throughout development and aging, which leads to different crystallin levels in different regions of the lens since lens cells are retained throughout the lifespan of an organism. Changing crystallin expression may be vital for lens function, which depends on a smooth gradient of refractive index that corrects for spherical and chromatic aberration [11-13]. Age-related changes in crystallin expression have been well documented in mammals but are poorly understood in the zebrafish, which as an aquatic vertebrate has an even higher index of refraction in the lens than the mammal. In terrestrial species, the cornea contributes to image refraction at the air-cornea barrier while in aquatic species, the index of refraction of the cornea is almost identical to water so the lens is responsible for image refraction [14].

There are many similarities in the optical and biophysical properties of zebrafish and mammalian lenses including expression of many of the same crystallins. Both zebrafish and mammalian lenses contain αA- and αB-crystallins, although the zebrafish has a gene duplication in αB-crystallin resulting in the expression of both αBa- and αBb-crystallins [15-17]. The β-crystallin proteins are also similar between zebrafish and mammals, and it has been proposed that six β-crystallin genes are found in all vertebrates [18,19]. γ-Crystallins are more divergent. Humans and mice contain genes for γA- through γF-crystallins, although γD- and γF-crystallins are pseudogenes in humans and these are specific to terrestrial mammals. Both zebrafish and mammals express γN-and γS-crystallins, and zebrafish additionally have multiple members of the γM-crystallin family of aquatic crystallin in the lens [20-22].

While crystallin gene and protein expression have been examined in the adult zebrafish lens and a few additional embryonic crystallins have been identified, this report is the first systematic analysis of changing crystallin expression during development and aging. We used size exclusion chromatography (SEC) combined with linear trap quadrupole Fourier transform tandem mass spectrometry (LTQ-FT LC-MS/MS; rank-order shotgun) proteomics to analyze protein expression in the lenses of larval, juvenile, and adult zebrafish. Advanced shotgun proteomics techniques allowed the identification of parent proteins from individual peptides in a complex protein sample [23,24]. With mass accuracies below 5 parts-per-million, shotgun proteomics is more sensitive than two dimensional (2D) polyacryamide gel electrophoresis for separation and detection of proteins with low abundance [25]. As expected, SEC and proteomics were consistent in the demonstration of varying levels of protein expression with age, especially among the crystallins. α-Crystallins, previously shown to have low abundance in the zebrafish lens, were found to increase dramatically during maturation and aging. Shotgun proteomics also identified novel crystallin peptides in the zebrafish lens that confirmed the existence of hypothetical crystallins in the zebrafish genome.

## Methods

### Lens homogenization

Fish were housed at 28.5 °C on a 14/10 h light/dark cycle and cared for in accordance with the University of Washington Institutional Animal Care and Use Committee. Lenses were dissected from WIK wild-type zebrafish, euthanized in 0.2 mM tricaine solution at 4.5 days (50 lenses), 10 days (50 lenses), three weeks (20 lenses), six weeks (20 lenses), six months (4 lenses), and 2.5 years (4 lenses) of age. All lenses appeared to be transparent. Fresh lenses were homogenized in 20 mM Tris-HCl and 1 mM EDTA, pH 8.0 on ice. After homogenization, phenylmethylsulfonyl fluoride (PMSF) was added to the solution to yield a final concentration of 0.1 mM. Homogenized lenses were immediately prepared for mass spectrometric analysis or analyzed by size exclusion chromatography.

### Size exclusion chromatography

Lens homogenates were separated into major protein components using a Biosep SEC-S3000 column (Phenomenex, Torrance, CA) and an ÄKTApurifier™ fast protein liquid chromatography (FLPC) (Amersham Biosciences, Pittsburgh, PA). A 50 μl sample of zebrafish lens homogenate (~2 mg/ml protein) was injected onto the column. The sample was eluted using 20 mM Tris-HCl, pH 8.0 at a flow rate of 0.5 ml/min. Protein elution was measured by absorbance at 280 nm. Fractions were collected every 250 μl, and select fractions were prepared for analysis using mass spectrometry. Each chromatogram was run at least three times. Individual molecular weight standards from the Gel Filtration Calibration Kit (GE Healthcare, Buckinghamshire, UK) were run to calculate fraction size. Protein concentration was calculated for each fraction from the six-week lenses using a bicinchoninic acid assay (BCA) protein assay kit (Thermo Scientific, Waltham, MA).

### Mass spectrometric analysis

Mass spectrometry was used to identify and quantify proteins present in zebrafish lens homogenates and select SEC fractions. Fifty microliters of sample was mixed with 50 μl of 12 M urea, 100 mM NH_4_HCO_3_, 7 μl of 1.5 M Tris-HCl pH 8.0, and 2.5 μl of 200 mM tris(2-carboxyethyl)-phosphine (TCEP). The sample was allowed to incubate at 37 °C for 1 h. Next, 20 μl of 200 mM iodoacetamide was added, and the sample was incubated for 1 h at 22 °C in the dark. After incubation, 4 μl of 1 M dithiothreitol (DTT) was added to the sample to react with excess iodoacetamide and incubated for 1 h at 22 °C. The sample was then mixed with 800 μl of 25 mM NH_4_HCO_3_ and 200 μl of methanol. One microliter of 1 mg/ml sequencing grade trypsin (Promega, Madison, WI) was added to the sample and allowed to incubate at 22 °C for 16 h. The sample was dried and dissolved in 190 μl of 5% acetonitrile (ACN) and 0.1% trifluoroacetic acid (TFA). The sample was loaded onto a pre-equilibrated UltraMicro Spin C18 column (Nest Group, Southborough, MA) for desalting. Peptides were eluted from the column using 80% acetonitrile (ACN) and 0.1% TFA. The peptide sample was dried and dissolved in 100 μl of 5% acetonitrile (ACN) and 0.1% formic acid.

Peptides were subjected to collision induced dissociation (CID) during LTQ-FT LC-MS/MS (Thermo Scientific) analysis to generate peptide tandem mass spectra (known as shotgun proteomics). Gas phase fractionation (GPF) was used to increase both individual protein sequence and proteome coverage [26]. For quantification with GPF analysis, data was acquired in quadruplicate. Each data set had four sets of identical stage 1 mass spectrometry (MS1) data from which “peptide quantity” was derived and four sets of unique stage 2 mass spectrometry (MS2) data sets from which peptide sequences were derived. With GPF, the MS2 data was acquired from four unique mass-to-charge ratio (m/z) ranges (400–600, 600–800, 800–1200, and 1200–2000), while the MS1 data was always acquired from the 400–2000 m/z range to provide the statistical significance needed for quantification. The software SEQUEST (Thermo Scientific) generated peptide sequence matches and identified parent proteins based on the International Protein Index (IPI). The algorithms, Peptide-Prophet and Protein-Prophet, used statistical routines to assign probability scores to the peptide sequence best fit and the likelihood that the parent protein was present [27,28]. Only proteins with a statistical probability score greater than or equal to 0.9 were included in the analysis. Spectral counting was used to calculate rank order from a single sample [29].

### Bioinformatics

Protein amino acid sequences were obtained from the IPI and NCBI. Multiple sequence alignments were performed using ClustalW [30]. Phylogenetic analyses were conducted using amino acid alignments with the neighbor-joining method (1000 bootstraps) in MEGA version 4 [31].

## Results

### Size exclusion chromatography

**Figure 1:**
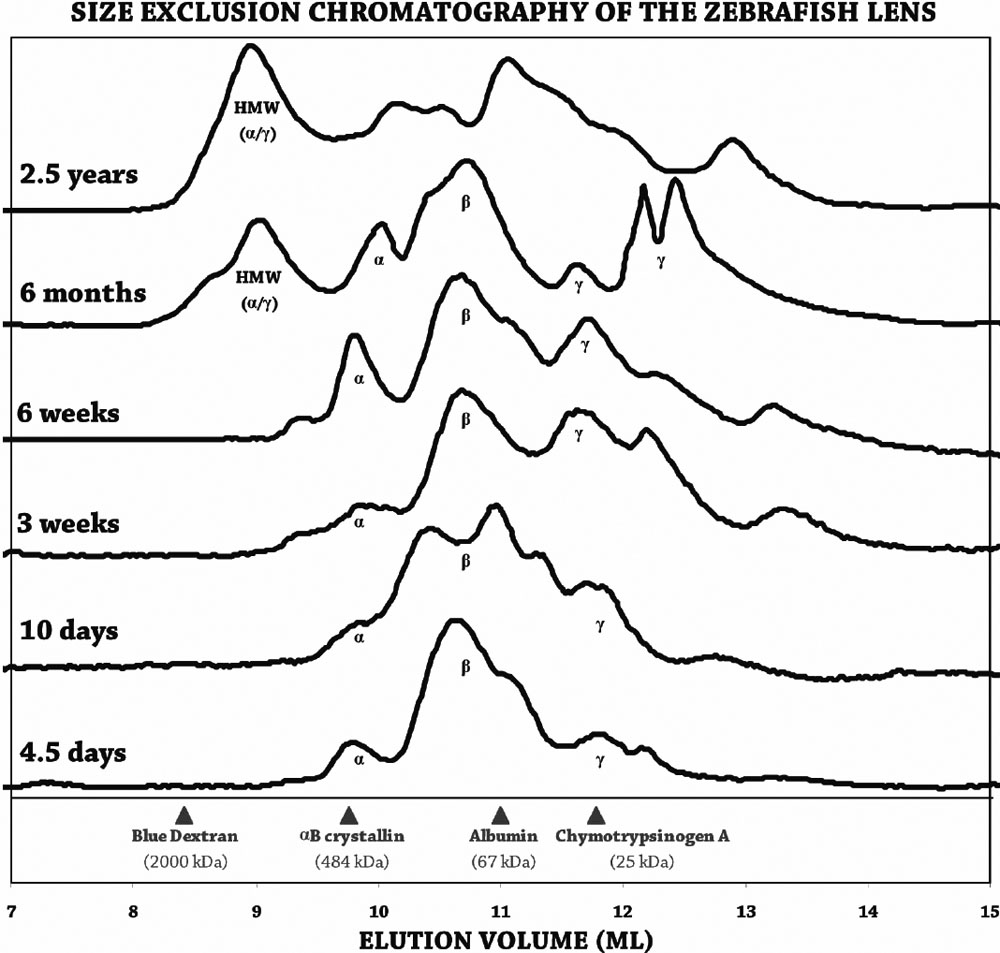
Size exclusion chromatography of zebrafish lens homogenates during development and aging. Absorbance at 280 nm for detection of proteins was plotted versus elution volume (x-axis), which corresponds with molecular size. Individual protein molecular weight standards are shown at the bottom of the graph. High molecular weight aggregates elute early followed by a broad peak of polydisperse α-crystallin oligomers with an average size of 24 subunits. Next, a broad peak of β-crystallin elutes from the column, which forms octamers, tetramers, and dimers, and finally, γ-crystallins, which are monomeric, were observed. The youngest lenses (4.5 days) were dominated by a large broad β-crystallin peak. α-Crystallin and γ-crystallin abundance increased during lens maturation, and a high molecular weight peak, first observed at six months, increased with age to become the largest peak by 2.5 years.

Size exclusion chromatography of the whole lens homogenates from WIK wild-type zebrafish determined the differences in major protein components in the larval (4.5 days, 10 days, 3 weeks), juvenile (6 weeks), adult (6 months), and aged (2.5 years) time points (Figure 1). Purified human αB-crystallin eluted from the column at 9.76 ml. Selected fractions from the six-month old lenses were analyzed by rank-order shotgun proteomics to confirm the protein composition of the predicted β-crystallin, γ-crystallin, and high molecular weight peaks (Table 1). The β-crystallin peak fraction (10.50–10.75 ml) contained only β-crystallins. The γ-crystallin peak fraction (12.50–12.75 ml) contained five different γ-crystallins and βB2-crystallin, which was also present in the β-crystallin peak. The high molecular weight peak fraction (9.25–9.50 ml) contained all three α-crystallins present in the fish lens as well as three γ-crystallins, which were not observed in the γ-crystallin peak. The shotgun proteomics confirmed that the major components in zebrafish lens crystallins separated by size exclusion chromatography into α-, β-, and γ-crystallin and high molecular weight peaks as observed in mammalian species.

**Table 1:** The three major peaks in the SEC of the six-month-old lenses were analyzed by shotgun proteomics to confirm protein content.

**Rank**	**HMW fraction 9.25–9.5 ml**	**IPI**	**β fraction 10.5–10.75 ml**	**IPI**	**γ fraction 12.5–12.75 ml**	**IPI**
1	αA-crystallin	509939.2	βB2-crystallin	501506.3	γS2-crystallin	868287.1
2	γM3-crystallin	607324.4	βA1a-crystallin	502528.2	γS4-crystallin	486227.2
3	αBa-crystallin	482033.2	βB3-crystallin	607344.1	γS3-crystallin	500990.2
4	γM2b-crystallin	504980.1	βA2-2-crystallin	513173.2	γS1-crystallin	495605.2
5	αBb-crystallin	488884.1	βA4-crystallin	490966.2	βB2-crystallin	501506.3
6	γM2a-crystallin	607295.1	βB1-crystallin	502990.3	γM7-crystallin	509894.2

The six most abundant proteins detected were crystallins. The high molecular weight (HMW) fraction contained α- and γ-crystallins, the β fraction contained only β-crystallins, and the γ fraction contained five γ-crystallins and one β-crystallin. IPI refers to the International Protein Index reference number.

In the homogenate of the 4.5-day lens, three broad protein peaks were observed, a small α-crystallin peak (9.78 ml), a large β-crystallin peak (10.62 ml), and a small γ-crystallin peak (11.79 ml; Figure 1). The α-crystallin peak remained small throughout the larval stage while the γ-crystallin peak increased progressively at 10 days and three weeks. At six weeks when zebrafish reach the juvenile stage, the α-crystallin protein fractions (9.00–10.25 ml) increased dramatically to approximately 22% of the total protein concentration. At this stage, the β-crystallin fractions (10.25–11.50 ml) were approximately 36% and the γ-crystallin fractions (11.50–13.50 ml) were 42% of the total protein. No high molecular weight peak was present up to six weeks, suggesting that the juvenile zebrafish lens contained undetectable levels of high molecular weight protein aggregates.

**Figure 2:**
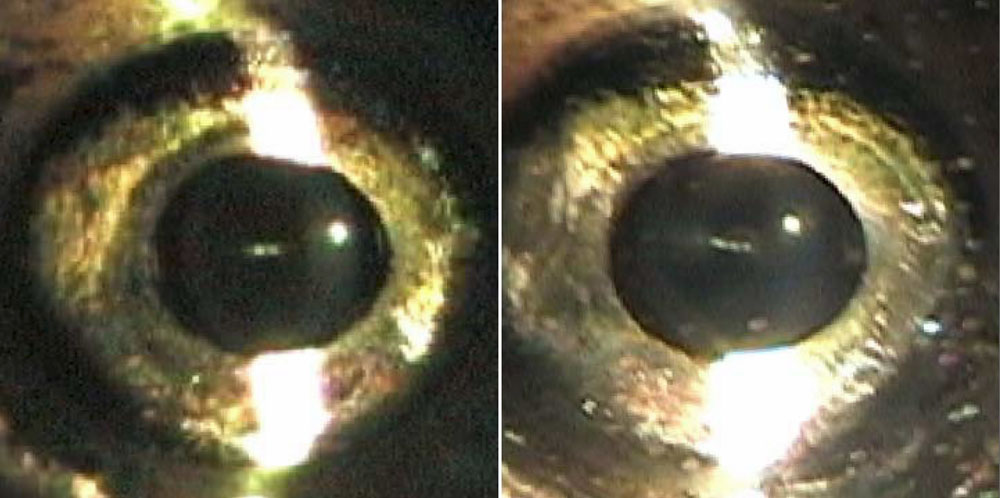
Slit-lamp views of living, anesthetized six-month-old (left panel) and 2.5-year-old (right panel) WIK zebrafish. Minimal light scattering is visible from the cornea, and no light scatter is visible from the lens. Lens transparency was maintained over 2.5 years, demonstrating the clarity of the lens and cornea in the zebrafish at ages up to 2.5 years.

By six months of age, a significant high molecular weight peak (9.01 ml) was observed (Figure 1). The broad α-crystallin (10.01 ml) and β-crystallin (10.73 ml) peaks remained well defined. The γ-crystallin peak observed at 11.62 ml appeared smaller than the corresponding peak in the six-week profile, and two new, smaller molecular weight γ-crystallin peaks (12.18 and 12.43 ml) were present. These peaks could represent the expression of different γ-crystallin proteins in the adult zebrafish lens or truncation products, which had not yet aggregated. When the 2.5-year-old, aged lens homogenate was separated using SEC, a high molecular weight peak (8.96 ml) was observed, although the 2.5-year-old lenses remained completely transparent by slit-lamp examination (Figure 2) and microscopy after removal (not shown).

### LTQ-FT LC-MS/MS proteomics: crystallins

Trypsin-digested peptides from zebrafish whole-lens homogenates were analyzed in quadruplicate by shotgun proteomics to generate a rank-order list of detectable proteins. The total number of proteins detected was 106 in the 4.5-day-old lenses (Appendix 1), 112 in the three-week-old lenses (Appendix 2), 136 in the six-week-old lenses (Appendix 3), and 234 in the six-month-old lenses (Appendix 4). In each age group examined, crystallins comprised the top 12 most abundant proteins on a rank-order list. Of the 37 embryonic and adult zebrafish lens crystallins reported previously, only two of the embryonic γ-crystallins (γM2d3- and γM2d4-crystallin) were not detected at any age in our analysis.

α-Crystallin proteins increased in abundance during maturation and aging (Table 2), similar to the SEC results. α-A-crystallin was the 28th most abundant protein at both 4.5 days and three weeks and increased to become the most abundant protein by six months. Neither αBa- nor αBb-crystallins were detected in the 4.5-day-old or three-week-old lenses while both were abundant in the six-month-old lenses.

**Table 2:** The change in α- and β-crystallin proteins with age was determined in zebrafish lenses by shotgun proteomics analysis.

**Crystallin protein**	**Rank order (relative abundance)**				**Chromosome**	**IPI**
	**4.5 days**	**3 weeks**	**6 weeks**	**6 months**		
αA	28	28	13	1	1	IPI00509939.2
αBa	-	-	-	9	15	IPI00482033.2
αBb	-	-	80	26	5	IPI00488884.1
βA1a	-	-	27	177	15	IPI00502528.2
βA1b	10	10	4	25	21	IPI00503999.3
βA2–1	7	14	9	11	6	IPI00495820.1
βA2–2	8	9	8	8	9	IPI00513173.2
βA4	9	8	1	10	19	IPI00490966.2
βB1	1	1	-	5	10	IPI00502990.4
βB2	-	24	15	4	8	IPI00501506.3
βB3	-	-	54	2	5	IPI00607344.1
βγX	83	-	71	82	7	IPI00493885.5
βA1c predicted (“βA1c”)	63	36	26	20	1	IPI00503128.2
βA1 crystallin, like (“βA1d”)”	4	6	7	32	14	IPI00504818.3
LOC553473 (“βB1b”)	2	2	2	57	14	IPI00607401.3
zgc:171773 (“βB1c”)	3	4	3	6	1	IPI00859087.1
zgc:171636 (“βB1d”)	-	-	76	80	15	IPI00858800.1

The numbers in columns 2–5 represent the rank order of protein abundance at each age listed (i.e., “1” indicates the most abundant protein detected in the lens). Column 6 “Chromosome” lists the chromosome from which the corresponding gene is transcribed. All three α-crystallins increased in abundance during lens maturation. β-Crystallins were abundant at all ages examined, and five novel β-crystallin proteins were identified. IPI refers to the International Protein Index reference number.

**Figure 3:**
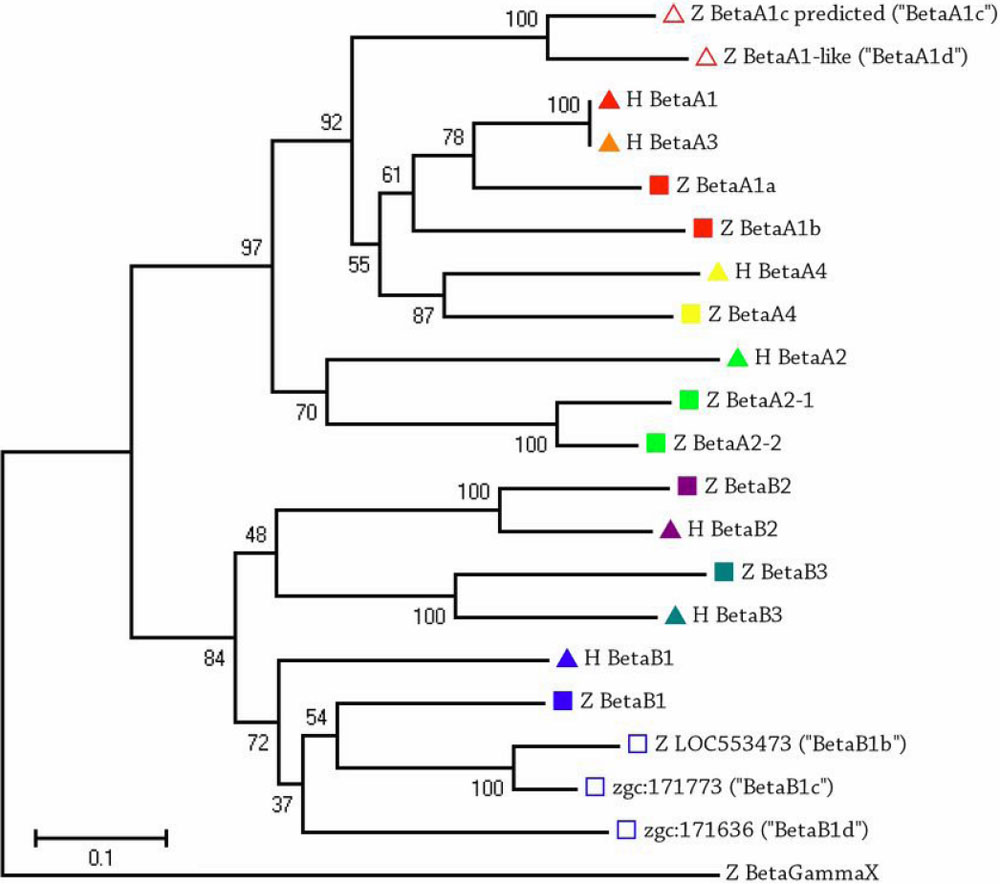
Phylogenetic tree of human and zebrafish β-crystallin genes constructed by Mega 4 with 1000 bootstraps. All zebrafish β-crystallins listed were detected by shotgun proteomics of the zebrafish lens (Table 2). Five novel β-crystallins (unfilled symbols) were detected and named based on their alignment. H, human; Z, zebrafish.

β-Crystallin proteins were frequently detected at all ages of lens examined (Table 2), consistent with SEC results. βB1-crystallin was the most abundant protein at 4.5 days and three weeks, and βA4-crystallin was the most abundant protein detected at six weeks. βB3-crystallin was the second most abundant protein at six months (behind αA-crystallin). Five novel β-crystallin proteins were detected in addition to the nine previously described β-crystallins (Table 2, Appendix 5). A phylogenetic analysis was conducted using the reported gene sequences for the six human β-crystallin genes, the nine previously reported zebrafish β-crystallin genes, and the five novel β-crystallin-like genes detected by shotgun proteomics (Figure 3). The two previously named “βA1c-crystallin predicted” and “βA1-like-crystallin” aligned with the βA-crystallin family genes, and these genes were re-titled βA1c- and βA1d-crystallins. Three novel proteins, zgc:171773, zgc:171636, and LOC553473, aligned closely with human and zebrafish βB1-crystallin. These three novel proteins have been titled βB1b-, βB1c-, and βB1d-crystallins. βB1b- and βB1c-crystallins were especially abundant in the juvenile zebrafish lens (Table 2).

**Figure 4:**
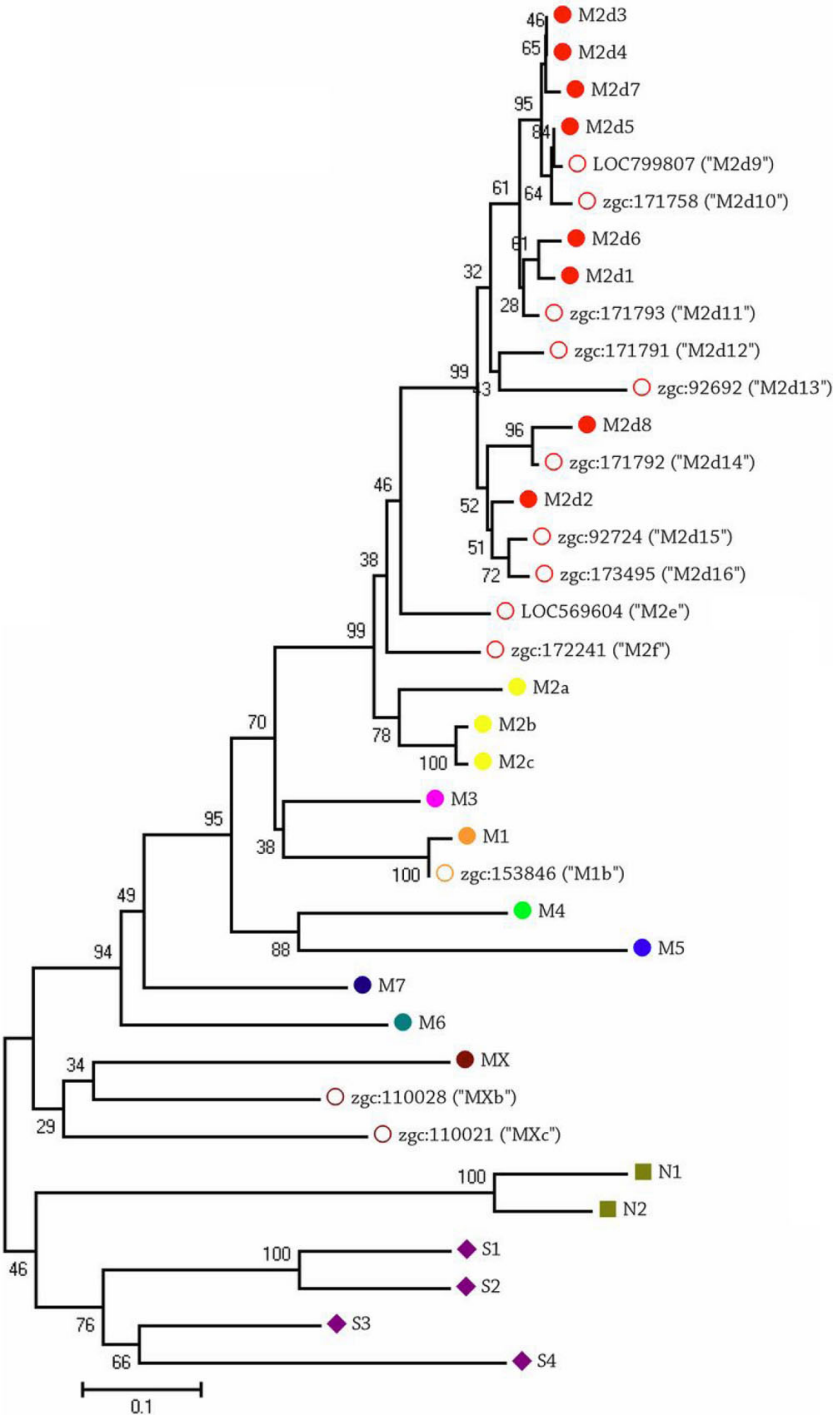
Phylogenetic tree of zebrafish γ-crystallin genes constructed by Mega 4 with 1000 bootstraps. All zebrafish γ-crystallins listed were detected by shotgun proteomics of the zebrafish lens (Table 3). Thirteen novel γ-crystallins (unfilled symbols) were detected and named based on their alignment.

**Figure 5:**
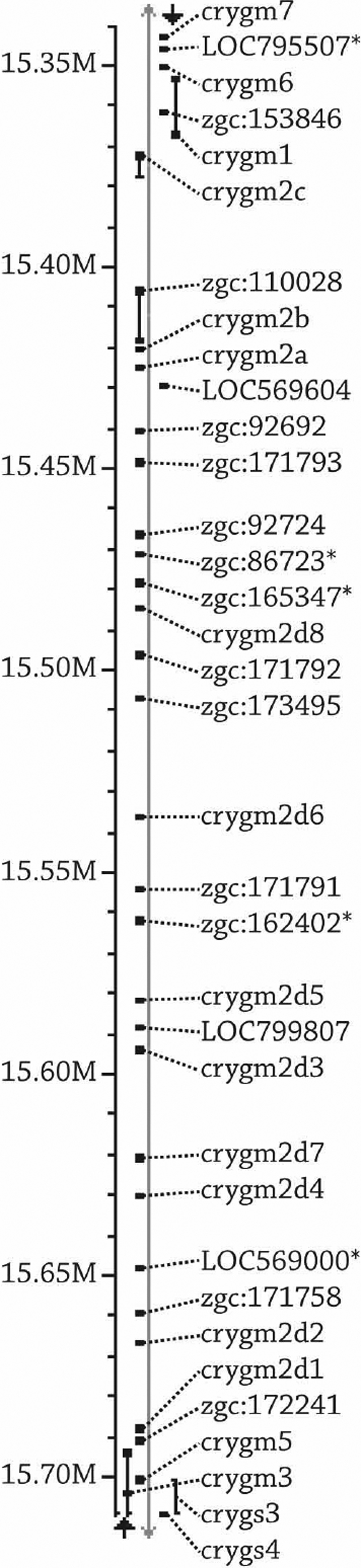
Zebrafish chromosome 9,400 kilobase-pair region containing 35 known and hypothetical γM-crystallin genes. Proteins from 30 genes in this region were found by shotgun proteomic analysis of the zebrafish lens (Table 3). The five genes marked (indicated by asterisk) were not detected but also show sequence similarity to the γM-crystallins. Gene positioning was determined by the NCBI map viewer, Ensembl Genes on Sequence Map. The scale on the left side of the image represents mega base-pairs. The gray line represents the chromosome. Genes on the left side of the gray line are located on the minus strand, and genes on the right side of the gray line are located on the plus strand.

Because so many different γ-crystallins were observed in the zebrafish lens, few γ-crystallins appeared in the top 10 most abundant proteins at any age. The exceptions to this were γMX-crystallin, which was abundant at all ages examined, γN2-crystallin, which was among the top five proteins in the larval and juvenile fish lens, and γS1-crystallin, which was the third most abundant protein in the six-month-old lenses (Table 3). Of the 24 γ-crystallins described previously, only five were detected in the 4.5-day-old lens while 18 different γ-crystallins were detected in the six-months-old lens. This result corresponded with the SEC data, which showed increasing γ-crystallin abundance during aging. In addition to the 24 γ-crystallins described previously, 13 novel γ-crystallin family proteins were detected in the zebrafish lens (Table 3, Appendix 5). A phylogenetic tree was constructed from the gene sequences of known and novel zebrafish γ-crystallin proteins (Figure 4). One gene (zgc:153846) aligned with γM1-crystallin and was titled γM1b-crystallin. Two genes (zgc:110028 and 110021) aligned with γMX-crystallin and were titled γMXb- and γMXc-crystallins. The remaining 10 novel γ-crystallins all aligned with the γM2-crystallin family and were named accordingly, γM2d9–16-, γM2e-, and γM2f-crystallin. The genes of 30 out of the 36 γ-crystallins detected in addition to five other γ-crystallin-like genes that were not detected were all located on chromosome 9 (Figure 5).

**Table 3:** The change in γ-crystallin proteins with age was determined in zebrafish lenses by shotgun proteomics analysis.

**Crystallin Protein**	**Rank order (relative abundance)**				**Chromosome**	**IPI**
	**4.5 days**	**3 weeks**	**6 weeks**	**6 months**		
γM1	-	32	32	14	9	IPI00495938.1
zgc:153846 (“γM1b”)	25	20	21	62	9	IPI00607433.4
γM2a	-	19	10	23	9	IPI00607295.1
γM2b	-	17	35	18	9	IPI00504980.1
γM2c	48	-	105	12	9	IPI00503886.1
γM2d1	-	-	-	206	9	IPI00485200.3
γM2d2	-	21	51	-	9	IPI00505178.4
γM2d5	-	-	-	185	9	IPI00614258.2
γM2d6	-	37	42	-	9	IPI00638856.2
γM2d7	93	99	122	-	9	IPI00486384.5
γM2d8	17	16	20	-	9	IPI00487422.2
LOC799807 (“γM2d9”)	15	-	52	-	9	IPI00835330.1
zgc:171758 (“γM2d10”)	26	29	40	74	9	IPI00859358.1
zgc:171793 (“γM2d11”)	-	40	37	-	9	IPI00866205.1
zgc:171791 (“γM2d12”)	12	11	12	226	9	IPI00833949.1
zgc:92692 (“γM2d13”)	13	12	17	108	9	IPI00502160.1
zgc:171792 (“γM2d14”)	16	-	24	-	9	IPI00863220.1
zgc:92724 (“γM2d15”)	18	25	29	63	9	IPI00774533.1
zgc:173495 (“γM2d16”)	-	74	95	-	9	IPI00866651.1
LOC569604 (“γM2e”)	-	15	11	46	9	IPI00613116.2
zgc:172241 (“γM2f”)	-	26	18	173	9	IPI00489442.3
γM3	-	84	22	21	9	IPI00607324.4
γM4	-	38	31	22	21	IPI00485316.1
γM5	-	-	47	41	9	IPI00483712.2
γM6	-	103	43	85	9	IPI00507423.3
γM7	-	68	28	27	9	IPI00509894.2
γMX	11	5	6	7	12	IPI00864931.1
zgc:110028 (“γMXb”)	-	88	48	166	9	IPI00503899.2
zgc:110021 (“γMXc”)	6	7	19	35	12	IPI00607474.1
γN1	-	27	14	13	2	IPI00499329.1
γN2	5	3	5	29	24	IPI00495773.1
γS1	-	-	93	3	22	IPI00495605.2
γS2	-	-	-	15	9	IPI00868287.1
γS3	-	-	-	28	9	IPI00500990.2
γS4	-	-	98	16	9	IPI00486227.2

The numbers in columns 2–5 represent the rank order of protein abundance at each age listed (i.e., “1” indicates the most abundant protein detected in the lens). Column 6 “Chromosome” lists the chromosome from which the corresponding gene is transcribed. Out of the total 36 γ-crystallin proteins identified, only 13 were detected at 4.5 days and 27 were detected at six months, indicating an increase in γ-crystallin abundance and diversity with aging. Thirteen novel γ-crystallins were identified. IPI refers to the International Protein Index reference number.

### LTQ-FT LC-MS/MS proteomics: non-crystallin proteins

Calpain 3 was the most abundant non-crystallin protein in the larval and juvenile zebrafish lens (Table 4). Calpain3 is a calcium-dependent protease involved in fiber cell differentiation [32]. The levels decreased from the 14th most abundant protein at 4.5 days to the 38th most abundant protein at six months of zebrafish development.

**Table 4:** Changes in non-crystallin proteins with age were determined in zebrafish lenses by shotgun proteomics analysis and selected abundant proteins are listed.

**Protein**	**Rank order (relative abundance)**				**Chromosome**	**IPI**
	**4.5 days**	**3 weeks**	**6 weeks**	**6 months**		
Calpain 3	14	13	16	38	17	IPI00489320.1
Major intrinsic protein of lens fiber 1	49	63	96	31	23	IPI00485872.1
Major intrinsic protein of lens fiber 2	32	47	126	76	23	IPI00607319.3
Grifin	-	34	36	42	3	IPI00506063.2
Lengsin	-	57	38	30	11	IPI00486208.6
Scinla	-	86	107	183	6	IPI00772565.3
Valosin-containing protein	20	33	34	44	5	IPI00505091.2
Thimet oligopeptidase 1	78	58	49	56	11	IPI00511724.1
β Actin 1	31	23	33	36	1	IPI00482295.2
Tubulin α2	36	56	99	-	11	IPI00488901.1
Tubulin β2B	54	69	-	232	5	IPI00494039.2
CP49/Bfsp2	-	79	25	17	2	IPI00512700.3
Vimentin	-	-	82	39	24	IPI00494222.1
Eukaryotic translation EF1-alpha	19	18	23	34	19	IPI00512240.1
Transketolase	35	35	-	141	23	IPI00498510.1
GAPDH	-	31	77	58	16	IPI00487455.2
Histone H2A-like	55	22	46	75	25	IPI00486495.1

The numbers in columns 2–5 represent the rank order of protein abundance at each age listed (i.e., “1” indicates the most abundant protein detected in the lens). The top six rows list non-crystallin proteins with its known function in the zebrafish lens. The middle section lists five cytoskeletal proteins, and the last four rows list ubiquitous proteins. IPI refers to the International Protein Index reference number.

The intermediate filaments, CP49 (Bfsp2) and vimentin, were not detected in the 4.5-day-old larval lens and increased in abundance during maturation. In the six-month-old fish lens, CP49 (Bfsp2), a lens-specific intermediate-filament, was the most abundant non-crystallin protein detected. Actin was abundant in the lens at all ages studied while tubulin α2 and tubulin β2b decreased in abundance during lens maturation. Several other cytoskeletal proteins were detected at low levels in the six-month-old lens including β spectrin, myosin, dynein, plectin, radixin, vinculin, actinin α1, and tubulin β5, β6, and α8 (Appendix 4).

The lens specific proteins, major intrinsic protein of the lens (Mip) 1 and Mip2, were detected at all ages studied. Mip2 was more abundant than Mip1 in the younger lenses while Mip1 abundance increased in the adult zebrafish lens. Three proteins known to be expressed in the zebrafish lens, Grifin, lengsin, and Scinla [33-35], were not detected in the 4.5-day-old larval lens and increased in abundance during maturation and aging. Two proteins associated with human familial Alzheimer disease were constitutively expressed at all ages, thimet oligopeptidase 1 and valosin-containing protein [36-39]. Several housekeeping proteins were detected at all ages examined, confirming that the shotgun proteomics method is a sensitive and effective method of protein detection and analysis.

Ribosomal proteins comprised 37% (39/106) of the total proteins detected in the 4.5-day-old lens (Appendix 1). At three weeks, 26% (29/112) of the total proteins were ribosomal (Appendix 2), and at six weeks, ribosomal proteins decreased to 11% (15/136; Appendix 3). At six months, ribosomal proteins comprised only 8% (18/234) of the total detectable lens proteins (Appendix 4). The decreasing abundance of ribosomal proteins may correlate with decreasing translation of new lens proteins with age and may also protect lens cells against deleterious effects of aging.

## Discussion

Rank-order shotgun proteomics combined with size exclusion chromatography was used to determine developmental changes in crystallin and non-crystallin proteins in the larval, juvenile, and adult zebrafish lens. α-Crystallin and γ-crystallins increased in abundance with lens maturation while β-crystallin remained abundant at all ages studied. Eighteen novel zebrafish crystallin proteins were identified.

The earliest report of zebrafish αA-crystallin transcripts was in the 24 hours post-fertilization (hpf) lens [40,41], and the αA-crystallin promoter was shown to drive lens expression of a green fluorescent protein (GFP) transgene starting at 25 hpf [15]. Neither αBa- nor αBb-crystallin transcripts were detected up to 48 hpf in whole zebrafish [40,41], although an αBa-crystallin polyclonal antibody was reported to stain the lens, retina, and brain at 24, 48, and 72 hpf [42] so the onset of αB-crystallin expression remains to be clarified. αB-crystallin transcripts were easily detected in the adult zebrafish lens [16,17]. In the 4.5 days post-fertilization (dpf) larval lens, the αA- but not αB-crystallin was detected and increased in both the α-crystallin peak fraction and the high molecular weight fraction during maturation and aging. α-Crystallins are vital for the development and maintenance of lens transparency and protect against protein unfolding and aggregation that lead to lens opacity [4-6,8,43]. The presence of all three α-crystallins plus γ-crystallins in the high molecular weight peak fraction at six months (Table 1) was expected because α-crystallin acts as a molecular chaperone to prevent γ-crystallin aggregation during aging [17,22,44,45]. While the current study reported α-crystallin to be as high as 22%, a previous study reported that α-crystallin comprised only 7.8% of the total zebrafish lens protein [19]. The difference may be related to the age of the fish in which α-crystallin content was measured. The 2.5-year-old zebrafish lenses examined in this study were completely transparent so it would be surprising if the adult zebrafish lens contained less than 10% of α-crystallin. Rodents, which have a similar life-span to zebrafish, have about 21.5% of α-crystallin in their lenses at six weeks [46], which is very similar to our observation that α-crystallins comprised 22% of total protein in the six-week-old zebrafish lens. The total amount of α-crystallin combined with its dramatic increase during lens maturation is consistent with the importance of α-crystallin in its protection against lens opacification during aging [4,8,47].

βB1-crystallin was the most abundant protein in the zebrafish lens at both 4.5 days and three weeks. Size exclusion chromatography results demonstrated that total β-crystallin content was much higher than α- or γ-crystallin in the larval zebrafish lens. βB1-crystallin transcripts were first detected in the zebrafish lens at 20 hpf, making it the earliest reported zebrafish lens crystallin [18]. In contrast to the zebrafish, βB1-crystallin is negligible in the embryonic mouse lens and sharply upregulates at birth, becoming the most abundant β-crystallin in the mouse by six weeks of age [48]. Analysis of rodent lenses during maturation detected a high percentage of γ-crystallin in the newborn lens with increasing α- and β-crystallins over the next few weeks [46,49]. γ-Crystallins were dominant in the embryonic dogfish lens [50]. Finally, newborn human lenses contain 35% α-crystallin, 40% β-crystallin, and 25% γ-crystallin [51]. While there are similarities in protein content of vertebrate lenses, there are differences in the timing of the expression of α-, β-, and γ-crystallins.

This study identified novel zebrafish crystallins. Eight “embryonic” γ-crystallins, γM2d1- through γM2d8-crystallin, were identified in normal 2 dpf embryonic lenses, which had not been previously detected in the adult lens [22]. In the 4.5 dpf larval lens, we detected only two of these proteins, γM2d7- and γM2d8-crystallin, as well as six other novel γM2d-crystallin-family members. All but two of the “embryonic” γ-crystallins, γM2d3- and γM2d4-crystallin, were detected in at least one of the time points measured in addition to eight novel γM2d-crystallin-family members, γM2d9- through γM2d16-crystallin. Because none of the γ-crystallins were abundant in the six-month old lens and many of them were abundant in the six-week-old lenses, it would be most accurate to refer to γM2d1- through γM2d16-crystallin as embryonic and juvenile crystallins. Genes for all of these crystallins were found on chromosome 9. In contrast, the four γS-crystallins were abundant in the six-month-old lenses and rarely found in younger lenses so these could be considered adult crystallins. The more divergent γN1-, γN2-, and γMX-crystallins had a more stable expression pattern and were moderately abundant at all ages examined. Even though zebrafish are known to have frequent gene duplications like αBa- and αBb-crystallins, 36+ γ-crystallins were a surprisingly large number, and a large percentage of these genes were found to be on chromosome 9 within a 400 kilobase-pair sized region of the gene (0.74% of the total chromosome 9 length; Figure 4). The functional purpose for so many γ-crystallin proteins in the zebrafish lens remains to be determined, especially because the non-refractive role of γ-crystallin is poorly understood.

The total number of ribosomal subunit proteins detected in the lens decreased dramatically during lens maturation, which would be expected due to the large decrease in the need to translate new proteins over time. The observed decrease in ribosomal proteins may also serve to protect the lens from aging as decreases in expression of the 60S ribosomal subunit has been correlated with increased cell survival [52-54].

The development and maintenance of lens transparency is especially important for zebrafish, which are visual hunters. In contrast, mice rely on other senses for obtaining food. The optical and biochemical similarities with the human lens and the experimental advantages of external lens development make the zebrafish a valuable model for studies of the lens during eye development and aging, which are currently conducted in several prominent zebrafish laboratories [19,20,55-58]. The results reported in the current study detail crystallin protein expression throughout zebrafish lens maturation and aging and provide a foundation for future systematic studies of the functional importance of crystallins in the development and maintenance of lens transparency and refraction in the vertebrate lens.

## Appendix 1. Shotgun proteomics results from 4.5-day-old larval zebrafish lenses.

To access the table, click or select the words “Appendix 1.” This will initiate the download of a pdf archive that contains the table. The rank lists the order of abundance. Spectral count indicates the number of times a peptide from the parent protein was detected in the sample and was used to generate the rank order.

## Appendix 2. Shotgun proteomics results from three-week-old larval zebrafish lenses.

To access the table, click or select the words “Appendix 2.” This will initiate the download of a pdf archive that contains the table. The rank lists the order of abundance. Spectral count indicates the number of times a peptide from the parent protein was detected in the sample and was used to generate the rank order.

## Appendix 3. Shotgun proteomics results from six-week-old juvenile zebrafish lenses.

To access the table, click or select the words “Appendix 3.” This will initiate the download of a pdf archive that contains the table. The rank lists the order of abundance. Spectral count indicates the number of times a peptide from the parent protein was detected in the sample and was used to generate the rank order.

## Appendix 4. Shotgun proteomics results from six-month-old adult zebrafish lenses.

To access the table, click or select the words “Appendix 4.” This will initiate the download of a pdf archive that contains the table. The rank lists the order of abundance. Spectral count indicates the number of times a peptide from the parent protein was detected in the sample and was used to generate the rank order.

## Appendix 5. The percent coverage and the number of unique peptides identified for each of the 18 novel crystallin proteins are listed for each age at which the protein was identified.

To access the table, click or select the words “Appendix 5.” This will initiate the download of a pdf archive that contains the table. Percent coverage was determined by the number of amino acids in all of the peptides identified by shotgun proteomics for each protein divided by the total number of amino acids in the protein sequence. The number of unique peptides refers to the peptide sequences identified by shotgun proteomics that are unique to an individual parent protein sequence.
